# A cluster-randomized controlled trial of strategies to increase adolescents’ physical activity and motivation during physical education lessons: the Motivating Active Learning in Physical Education (MALP) trial

**DOI:** 10.1186/1471-2458-12-834

**Published:** 2012-10-01

**Authors:** Richard R Rosenkranz, David R Lubans, Louisa R Peralta, Andrew Bennie, Taren Sanders, Chris Lonsdale

**Affiliations:** 1 Kansas State University, Manhattan, USA; 2 University of Western Sydney, Penrith, Australia; 3 University of Newcastle, Newcastle, Australia

**Keywords:** Physical activity, Physical education, School, Children, Adolescent, Youth, Motivation, Self-determination theory

## Abstract

**Background:**

The physical activity (PA) levels of many children and adolescents in Australia are currently insufficient to promote health benefits. Physical education (PE) programs aim to promote PA and reach nearly all school-aged children, but PA levels within PE lessons are often low. PE teachers may influence children’s motivation to be physically active in PE lessons, but little is known about teacher strategies that effectively motivate children to participate in PA, and few intervention studies have examined motivational strategies in PE. The purpose of this study was to compare the effect of three motivational strategies, each based on Self-Determination Theory (SDT), on PA levels, and their hypothesized antecedents, during year 8 PE lessons.

**Methods/design:**

This study employed a cluster-randomized controlled trial design. Following a familiarization session, PA levels and hypothesized PA antecedents were measured during a baseline lesson and a post-intervention or control lesson. Teachers (n = 16) and their classes from five secondary schools in Sydney, Australia were randomly assigned into four blocks and instructed to provide one of four 20-min lesson teaching strategy conditions: (1) explaining the relevance of activities; (2) providing choice from PA options selected by the teacher; (3) providing equipment and free choice of activities; or (4) usual practice. The primary outcomes were lesson time spent in moderate-to-vigorous PA, and motivation towards the lesson. Secondary outcomes were perceptions of teacher behavior, psychological needs satisfaction, and lesson time spent in sedentary behavior. PA and sedentary behavior were measured during baseline and post-intervention lessons with waist-mounted Actigraph GT3X accelerometers. Teacher behavior, psychological needs satisfaction, and motivation were assessed via questionnaires at the end of each lesson. Linear mixed-model analyses will be run on all outcomes, with students nested within teachers as a random effect.

**Discussion:**

Study findings will inform the development of effective SDT-based teaching strategy interventions to enhance students’ psychological needs satisfaction, motivation, and PA levels. More effective teaching strategies may be identified that are capable of improving adolescents’ PA levels, and thereby provide beneficial population health outcomes.

**Trial registration:**

This trial is registered with Current Controlled Trials and is traceable as ISRCTN07038258.

## Background

Physical inactivity is one of the leading modifiable causes of death and disease in Australia [1]. Regular physical activity (PA) decreases the risk of developing cardiovascular disease, diabetes, some cancers, obesity, osteoporosis, and other chronic conditions [2], but many children and adolescents in Australia are not sufficiently active to accrue associated health benefits [3]. In response to this evidence and data showing rising levels of childhood and adolescent obesity [3], public health efforts have emphasized the importance of schools in the promotion of PA among youth [4-8].

Recently, the Australian Government-commissioned Crawford Report [9] described the central role of physical education (PE) programs for increasing the PA levels of youth. PE lessons reach the majority of school-aged youth, and therefore the promotion of PA through PE has far-reaching health implications for millions of Australian youth [9]. Students’ PA levels in PE lessons, however, are often very low [10]. To achieve increased PA both within and beyond PE lessons, it is important for school-aged youth to be sufficiently and appropriately motivated [11,12]. PE teachers can play an important role in motivating students to be physically active within [13], and beyond PE lessons [14]. Youth who lack motivation in PE often report negative experiences and relationships with their PE teachers [15], which is why it is imperative for researchers to examine teaching strategies for PE teachers to motivate their students more effectively toward achievement of higher levels of PA.

### Theoretical framework

The current study is based on Self-Determination Theory (SDT) [16,17] which has been widely applied to a variety of life contexts, including education [18], sport [19], exercise [20], and PE [11,21]. According to SDT tenets, social-contextual factors (e.g., teaching strategies used by PE teachers) can affect individuals’ motivation by satisfying (or undermining) three key psychological needs: 1) autonomy: the need to self-endorse activities and beliefs; 2) competence: the need to effectively interact with one’s environment and yield desirable outcomes; and 3) relatedness: the need to feel connected and accepted by significant others [16,17].

In the context of PE, when teachers use motivational strategies that satisfy key psychological needs, students will feel more self-determined to participate in PE, and thus will be more physically active during lessons [11]. Motivational strategies that support student needs include: (1) “choice”: providing students with the opportunity to make decisions about the activities they undertake during lessons; (2) “relevance”: providing a rationale and explaining to students the relevance of an activity; (3) “acknowledgement”: acknowledging students’ difficulties when learning skills; and (4) “feedback”: providing feedback using praise for students’ effort and improvement. Previous studies have shown that these strategies can satisfy the three key psychological needs in the PA context, and are essential to well-being, learning, and the development of autonomous forms of motivation (e.g., intrinsic motivation) [22-24].

In contrast, teachers’ motivational strategies that undermine student autonomy (e.g., controlling strategies such as discouragement of student initiative, overt teacher control, conditional student acceptance and praise) may thwart students’ psychological needs and thereby result in controlling forms of extrinsic motivation or lack of motivation, anxiety, and poor psychological adjustment [25]. In the PE context, such dysfunctional motivational strategies may reduce PA levels. Experimental research in the contexts of education [26] and PA promotion in adults [27] has supported these theoretical predictions and a recent study showed that providing children (aged 8–12) with choice increased physical activity in a laboratory setting [28].

Despite the apparent influential role of teachers’ motivational strategies, intervention studies to enhance teachers’ motivational strategies in PE are rare. Chatzisarantis and Hagger [29] evaluated the effects of an SDT-based PE intervention in which the five teachers in the experimental condition learned the four aforementioned motivational strategies, and five teachers in the comparison condition learned only the “relevance” and “feedback” strategies. Students in the experimental condition, whose teachers had also learned the “choice” and “acknowledgement” strategies, reported greater self-determined motivation toward PE and more leisure time PA than comparison participants. Although these results are encouraging, limitations of the study include: (1) the lack of measurement of PA within the PE lesson and the reliance on self-report PA measures in leisure time; (2) the lack of objective assessment of teacher behaviors leading to an inability to determine to what degree each of the strategies was employed by teachers; and (3) the inability to determine which of the four strategies influenced student motivation. Indeed, Chatzisarantis and Hagger [29] investigated the additive effect of “choice” and “acknowledgement” to “relevance” and “feedback”, but no comparison was made with teachers’ usual teaching style.

In a recent experimental study, Cheon et al. [30] investigated the effect of an SDT-based training program for PE teachers on student motivation and intentions to be physically active outside school. Training involved five hours of multi-media presentations and discussion where teachers learned four types of SDT-based motivational strategies. Compared with controls, students whose teachers had participated in this training reported greater increases in needs satisfaction, self-determined motivation, and intentions to be physically active outside school. Once again, these findings are promising, but the effect of autonomy support on PA during PE lessons was not assessed, and only PA intentions, not actual PA behavior, was measured. Also, Cheon et al. [30] reported that all four types of motivational strategies were implemented by teachers, but the study design did not allow the authors to assess the extent to which each strategy influenced student motivation and other outcomes. Indeed, all teachers learned all four strategies and, thus, it is unknown if some strategies were more effective than others.

In the present study, we addressed a limitation of the Chatzisarantis and Hagger [29] and Cheon et al. [30] studies by employing an objective measure of PA within PE lessons. We also examined the independent effects of different motivational teaching strategies. Specifically, we compared strategies designed to explain relevance and provide choice.

Explaining the relevance of activities and providing opportunities for students to make choices are considered important strategies to promote autonomous student motivation [31]. In academic classrooms, there is evidence that explaining relevance may have a greater impact on student motivation and engagement than providing choice [31,32]. To the authors’ knowledge, no PE-based research has investigated the importance of explaining relevance to students. However, the influence of choice in PE lessons has received some attention. Ward and colleagues [33] investigated the influence of providing adolescent girls with a single choice per PE lesson. They found that this strategy increased self-determined motivation, but did not influence PA levels during PE lessons (as measured by pedometers). It is possible, however, that Ward et al’s [33] decision to only provide one opportunity for choice during a lesson may have minimized treatment effects. Indeed, a meta-analysis [34] found that providing two to four opportunities for choice had the greatest effect on motivation. Lonsdale et al. [11] compared student PA during structured lessons (no choice) and complete free choice periods, during which equipment was provided, but students were able to decide for themselves what activities they undertook. This manipulation resulted in greater PA during the free choice period. Neither Ward et al. [33] nor Lonsdale et al. [11] investigated varying levels of choice or the effect of any other autonomy supportive strategy (e.g., explaining relevance) on students’ need satisfaction, motivation, or PA levels. As noted, no research has investigated the independent effects of these strategies on PA in PE lessons. In the present study, we examined these independent effects by comparing the effectiveness of: (1) “relevance” - explaining the relevance of activities; (2) “providing choice” - providing a number of PA options selected by the teacher; (3) "free choice” – providing complete free choice of activities.

### Aims of the study

The aim of the current project was to investigate the relative effects of three motivational teaching strategies on motivation and PA levels during Year 8 PE lessons. Specifically, we sought to use a cluster-randomized controlled trial to test the effectiveness of three SDT-based motivational teaching strategies, in comparison with usual teaching practices. Understanding which strategies have the greatest impact on PA may allow for the development of effective interventions to increase long-term student motivation and PA during PE lessons, and potentially beyond PE.

There were three main research questions:

Research Question 1: Do students differ across the three motivational teaching strategy conditions and control (usual practice) teaching condition with regard to moderate-to-vigorous PA (MVPA) (primary outcome) and sedentary behavior (secondary outcome) during PE lessons?

Research Question 2: Do student motivation towards PE lessons (primary outcome), needs satisfaction, and perceptions of teacher behavior (secondary outcomes) differ across the three motivational teaching strategy conditions and the control (usual practice) teaching condition?

**Figure 1:**
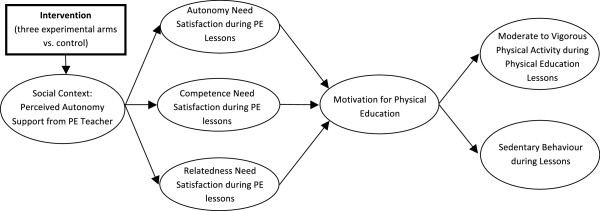
**Hypothesized relationships** .

Research Question 3: Are the effects of motivational teaching strategies on PA and sedentary behavior mediated by teachers’ autonomy supportive behavior and students’ needs satisfaction and motivation during lessons? (See Figure 1).

### Hypotheses

Compared with usual practice, we hypothesized that PE lessons in which teachers explained the relevance of activities and provided students with choices would:

1. show greater increase in the percentage of time students spend in moderate-to-vigorous physical activity during PE lessons.

2. show greater decrease in the percentage of PE lesson time that students spend in sedentary behavior.

3. show greater increase in students’ autonomous motivation during lessons.

4. show greater decrease in students’ controlled motivation during lessons.

5. show greater increase in students’ perceptions of autonomy, competence, and relatedness during lessons.

6. show greater increase in students’ and independent raters’ perceptions of teachers’ autonomy supportive behavior.

7. produce effects that would be by teachers’ autonomy supportive behavior and students’ needs satisfaction and motivation (see Figure 1).

## Methods/design

The Human Research Ethics Committee at the University of Western Sydney provided ethical approval for this cluster-randomized controlled trial. The study was conducted in Independent and Catholic schools in Sydney, New South Wales, Australia during 2011. Ten schools were identified using publicly available listings, and invited via telephone and email through publicly available contact details. The first five schools whose principal provided consent were included in the study. Once consent was also received from department heads and teachers of Year 8 PE classes, the research team met briefly with the students to provide basic information about the study. At this time, information sheets and consent forms were distributed to students. Students who were unable to participate in PE classes were not eligible to participate in the study. All other Year 8 PE students who returned signed parental consent forms and provided personal assent were allowed to participate in the study. Students who did not return consent forms still participated in the lessons alongside their peers, but did not wear accelerometers, or complete questionnaires.

**Figure 2:**
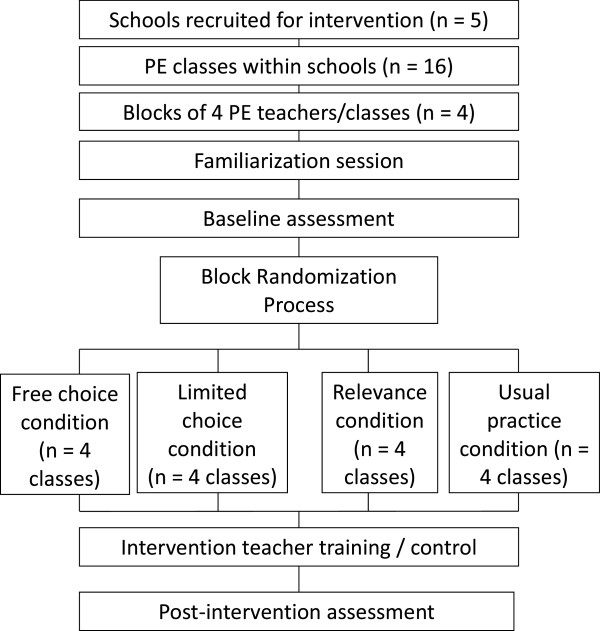
**MALP study flow chart** .

Figure 2 provides a summary of the study design, a cluster-randomized controlled trial with four study arms. To begin, a familiarization session was held during a PE lesson. During this lesson, students wore an accelerometer and teachers wore a microphone attached to their shirt that connected wirelessly to an audio recording device. The purpose of this session was to minimize potential reactivity to these monitoring devices during baseline and post-intervention sessions. Data collected during this familiarization lesson will not be analyzed. Baseline and post-intervention data were collected from all participating students, regardless of study arm, during and immediately after two 20-min segments of PE lessons. In between these two lessons, teachers received a brief intervention (three experimental arms) or were asked to continue with usual practice (control arm).

### Sample size and power calculation

Lonsdale et al. [11] found a large difference in PA levels between free choice and structured PE lesson conditions (Cohen’s *d* = 1.07). We adopted a conservative approach and estimated a moderate effect size (medium *f* = 0.25) for mixed (between and within-subjects) analysis. Based on this estimation, as well as an estimated correlation of *r* = 0.5 among the repeated measures of PA (*r* = .53 in a previous study [11]), we required a sample of 76 students in order to achieve 95% power, with alpha set at .05. Taking into account the clustered nature of the data, this sample size was multiplied by a correction factor of 1+(*m* - 1)ρ, called the design effect, where *m* is the average cluster size and ρ is the intra-class correlation coefficient [35]. Assuming an *m* of 25 students per class and ρ = 0.1 (estimated based on a previous study [36]), the correction factor is 3.4. As a result, a sample of 258 students (i.e., 76 × 3.4 = 258) was required. To allow for lack of informed parental consent or assent from some students, and to protect against participant attrition across the four teaching conditions, we attempted to increase the sample by 20% and recruit 308 students from 16 PE classes.

### Randomization

The 16 PE teachers, with their students, were randomly assigned to one of four conditions (three interventions and one control). An independent researcher, blinded to the study hypotheses, performed the allocation after the baseline assessment was administered. Randomization was conducted at the class-level and within schools to control for school characteristics, using a computerized random number generator with blocked randomization scheme (block size = 4). School 1 (Catholic – coeducational), School 2 (Independent – girls only), and School 3 (Independent – coeducational) each provided four classes of students and one class from each school was randomly assigned to one of the four conditions. School 4 and School 5 (two classes each) were both Catholic boys schools. Together, these two schools constituted the final block from which four classes were randomly assigned.

### Intervention and fidelity check

Prior to the post-intervention PE lesson, each teacher met with a researcher (CL) for approximately 20 minutes to be trained to deliver the assigned motivational strategy. This meeting took place 20–48 hours before the post-intervention lesson. During this meeting, the investigator asked the teacher to share the lesson plan from the baseline lesson or to describe the baseline lesson activities if the lesson plan was not available. The investigator asked the teacher to devise a lesson for the post-intervention PE lesson that was similar in structure to the baseline lesson. He then guided the teacher through a one-page outline of the proposed teaching strategy. This outline included the strategy name, definitions, the rationale behind the strategy, and guidelines for implementation. The teacher had the opportunity to ask questions, and the researcher and teacher discussed plans for strategy implementation in the upcoming lesson.

The researcher trained the teachers randomized to the “relevance” arm (n = 4) to make statements during the PE lesson that explained the rationale behind the activities in the lesson, and made it clear how the activities were relevant to students’ lives. The researcher trained the teachers randomized to the “providing choice” arm (n =4) to provide students with opportunities to make choices from options that were selected by the teacher. In line with meta-analytic evidence regarding the provision of choice [34], teachers were asked to provide between 2–4 opportunities for choice during the lesson. When providing students with an opportunity a choice, teachers were also asked to avoid overburdening students with too many options from which to choose (i.e., ≥ 5). The researcher asked the teachers randomized to “free choice” (n = 4) to provide the students with complete free choice; meaning that equipment would be provided, but the teacher would not provide any instruction. It could be argued that this strategy does not include adequate lesson structure and may undermine perceived competence [37]. Previous research, however, has indicated that complete free choice can increase students’ total PA accumulated during a lesson, relative to standard practice [11]. Therefore, an investigation of potential effects on needs satisfaction, motivation, moderate-to-vigorous PA and sedentary behavior is warranted. The researcher asked teachers randomized to the control condition (n = 4) to continue with their usual practice.

Teachers in all four study arms had their verbal communication audio recorded in baseline and post-intervention lessons. To test the fidelity of the interventions, two independent researchers assessed teachers’ provision of autonomy support. Specifically, the researchers used four items from the Teacher as Social Context Questionnaire [21] to rate the extent to which: (1) “The teacher gave the students choices about how they do the tasks in PE”; (2) “The teacher talks about how the students can use the things they learn in PE”; (3) “The teacher listens to the students’ ideas”. and (4) “It seems like the teacher is always telling the students what to do”.

As indicated in Table 1, compared with the control and “relevance” arms, teachers in the “providing choice” and “free choice” arms were expected to exhibit greater increases in provision of autonomy support from baseline to post-intervention on the first item. Teachers in the “relevance” arm were expected to exhibit larger increases than the other three arms on the second item. The raters hold PhD qualifications in a related discipline (e.g., psychology) and have knowledge of motivational theory applied to physical activity contexts, as evidenced by at least five peer-reviewed publications on the topic. Also, raters were blinded to study hypotheses and teachers’ allocation to the control or experimental arms.

**Table 1:** Motivation strategy conditions

**Intervention arms**	**Description**	**Hypothesized impact on PAand SDT constructs, relativeto baseline**
Free Choice	Equipment provided, but no instruction by the teacher took place.	Higher PA levels
		Lower sedentary behavior
		Higher autonomy support from teacher (especially perceived choice)
		Higher basic need satisfaction
		Higher self-determined motivation
Providing Choice	Provided options and offered opportunities for students to take initiative.	Higher PA levels
		Lower sedentary behavior
		Higher autonomy support from teacher (especially perceived choice)
		Higher basic need satisfaction
		Higher self-determined motivation
Relevance	Provided rationale and explained relevance of activities	Higher PA levels
		Lower sedentary behavior
		Higher autonomy support from teacher (especially perceived relevance)
		Higher basic need satisfaction
		Higher self-determined motivation
Usual practice	No intervention. Lesson activities conducted without researcher-directed teaching strategies	No change in PA levels
		No change in sedentary behavior
		No change in basic need satisfaction
		No change in self-determined motivation

Note: PA = physical activity, SDT = Self-Determination Theory.

### Outcome measures

Different schools and classes were expected to have PE lessons of varying duration and, due to fatigue, it is possible that MVPA levels at the end of longer lessons could be lower than earlier in the lesson. To standardize across lessons of varying durations, all PA data collection occurred during the first 20 min of teaching time during each lesson for the baseline and post-intervention assessments (i.e., after students had changed their clothes and been fitted with an accelerometer). ActiGraph GT3X accelerometers (ActiGraph; Pensacola, FL) were used to assess PA levels (percentage of time spent in MVPA and percentage of time spent sedentary) of each student in this study. Monitors were synchronized with an external clock and initialized to start recording data in three axes of motion in 1-sec epochs, a minimum of 30 min before and after the scheduled PE lessons. Research assistants helped students place an Actigraph monitor, via adjustable elastic belt, over the right iliac crest, prior to the start of each observed PE lesson, to wear for the duration of lesson. Raw accelerometer counts were uploaded to a lab computer, and saved to a customized Microsoft Excel file. Data outside the recorded start and finish time for given sessions was disregarded. Data were checked for spurious values that did not coincide with the direct observation records; all data between start and finish times for all lessons were included in the analyses. Freedson’s MET prediction equation [38] was used to determine PA intensity, and 100 counts per minute was used as a criterion to determine sedentary time [39].

At the 20-min mark of the baseline and post-intervention lessons, each student completed the four items from the Teacher as Social Context Questionnaire described previously. This version of the questionnaire is designed to measure students’ perceptions of their teachers’ autonomy supportive behavior [21] (e.g., “The teacher gives us choices about how we do the things in today’s class”). Students also completed measures of autonomy need satisfaction [24,40], competence need satisfaction [41], relatedness need satisfaction [42], and controlled and autonomous forms of motivation (Situational Motivation Scale) [43]. Each measure has received empirical support regarding reliability and validity in English-speaking samples of this age group [11,14,21,41-44].

Research assistants, who were senior undergraduate students, conducted all data collection. Research assistants received two hours of data collection training from researchers (CL and RR) who had experience with these data collection techniques. All research assistants were blinded to study hypotheses and cluster allocation (i.e., to intervention or control). Students in each PE class were also blinded to allocation; however, teachers were necessarily un-blinded when they received the intervention (i.e., post-baseline assessment) or learned that they were in the control arm of the trial.

Students’ age, gender, place of birth (Australia or other) and main language spoken in the home (English or other) were collected. A proxy measure of socio-economic status was collected, as students reported their home postcode. This postcode was cross-referenced with census data [45] to determine the economic decile of each student’s home neighborhood.

### Adverse events

Wearing accelerometers and completing questionnaires pose little risk of harm to participants. Teaching strategies were based on theory and research evidence, indicating that they can effectively motivate behavior and no adverse events were reported.

### Planned statistical analysis

To assess the reliability of the teacher behavior ratings provided by the independent observers, intra-class correlation coefficients (ICCs) will be calculated. The fidelity of the interventions will be assessed using a MANOVA (with follow-up comparisons). If the manipulation of teacher strategies was successful, independent observer ratings of the intended strategy for each condition should be higher than ratings in other conditions. For example, ratings of choice should be higher in the two choice conditions than in any of the relevance and control conditions. To assess differences in primary and secondary outcome variables, linear mixed model analyses will be run on accelerometer counts, motivation and basic needs satisfaction, with students nested within teachers as a random effect. All analyses will be conducted based on the intention-to-treat principle.

## Discussion

This cluster randomized controlled trial was designed to evaluate potentially effective motivational teaching strategies in PE with the aim of helping adolescents to have more self-determined motivation for PA, and to be more physically active, both in PE lessons, and beyond. The study design will allow us to compare the effect of three Self-Determination Theory-based teacher motivational strategies on PA levels, motivation, and basic needs satisfaction during PE lessons. The findings from this study will be used to inform effective SDT-based teaching interventions to promote PA in youth.

Our study was among the first to use objective measures of PA along with assessments of PA antecedents in an experimental design that tests the relative effectiveness of Self-Determination Theory-based teaching strategies. Beyond novelty and rigor, strengths of this study include the use of a real-world setting in community-based PE lessons, with professional teachers implementing intervention strategies with their own students in their lessons. Also, the study’s manipulation checks will strengthen our ability to determine how well teachers implemented the intended interventions, which is crucial for making causal inference regarding the relationship between teaching strategies and student PA. An additional strength comes from the use of both “providing” and “free” choice teaching strategy conditions, which will enable us to determine the type or degree of choice needed to impact motivation and PA levels in adolescents.

Alongside study strengths, the following limitations should be noted. Our study sample was limited to teachers and students from Independent and Catholic schools, and results may not be applicable to government-funded school settings. Although our study sample size has been planned with adequate power to detect hypothesized relationships, generalizability to other Independent and Catholic schools could be limited both by the modest sample size and by potential selection bias stemming from the use of only schools whose principals were among the first to participate.

### Implications of study findings

At the conclusion of the study, researchers and policy makers will benefit from study findings in several ways. Foremost among them is the identification of more effective ways to motivate adolescent boys and girls toward being more physically active, which has great potential to improve health at the population level. In this case, researchers and policy makers will not only gain further understanding about which teaching strategies are most likely to result in higher PA levels, but will also gain understanding about need satisfaction and self-determined motivation, PA antecedents with potential to impact PA in a sustainable manner. Schools and school districts, potentially in conjunction with researchers, may make use of the information about motivational teaching strategies to plan professional development programs. Indeed, we expect that understanding how “relevance”, “providing choice,” and “free choice” influence motivation and physical activity will allow researchers and educators to design effective interventions. The long-term intervention effects on physical activity behaviors during PE lessons and outside school hours needs to be investigated.

## Competing interests

The authors declare that they have no competing interests.

## Authors’ contributions

CL, RR, and DL lead the development and design of the study, and will conduct data analysis. CL is the principal investigator, with DL and RR as co-investigators. CL was responsible for training teachers to deliver the motivational teaching strategies. LP and AB contributed to the design of the motivational teaching strategy interventions and helped coordinate data collection. TS was the project manager and was involved in study design. All authors contributed and have approved the final manuscript.

## Pre-publication history

The pre-publication history for this paper can be accessed here:

http://www.biomedcentral.com/1471-2458/12/834/prepub
